# Contrast-Enhanced Ultrasound Imaging of Prostate Gland in Neutered Dogs

**DOI:** 10.3390/ani11020559

**Published:** 2021-02-20

**Authors:** Stefano Spada, Gary C. W. England, Massimo Vignoli, Augusto Carluccio, Marco Russo

**Affiliations:** 1 Department of Veterinary Medicine and Animal Production, University of Naples, Federico II, 80137 Naples, Italy; 2 School of Veterinary Medicine and Science, Sutton Bonington Campus, University of Nottingham, Loughborough LE12 5RD, UK; gary.england@nottingham.ac.uk; 3 Faculty of Veterinary Medicine, University of Teramo Piano d’Accio, 64100 Teramo, Italy; mvignoli@unite.it (M.V.); acarluccio@unite.it (A.C.)

**Keywords:** contrast-enhanced ultrasound, B-mode ultrasound, prostate, neutered dogs

## Abstract

**Simple Summary:**

Diagnosis of prostatic neoplasia in dogs remains critical since none of the diagnostic methods routinely performed seem to have sufficient sensitivity or specificity. In recent years, contrast-enhanced ultrasound of the prostate gland has been successfully performed in intact dogs. However, data regarding the performance of the technique in castrated dogs have not been provided, despite the incidence of prostate neoplasia potentially being higher after castration. The purpose of the present study was to evaluate the application of contrast-enhanced ultrasound in castrated dogs and to describe the vascular pattern of the canine prostate. Prior to contrast-enhanced ultrasound evaluation, B-mode ultrasound was performed to assess prostate size and parenchymal features. In all cases, perfusion kinetics of the contrast agent within the prostate was analyzed, with the aim of detecting any areas with increased microvessel density. None of the dogs had any ultrasonographically detected pathology. Contrast ultrasound allowed an improved and enhanced ability to evaluate the prostate compared with B-mode ultrasound. This technique is not yet to become a first-choice diagnostic tool; however, since the results of this study are favorable, it seems likely that contrast ultrasound will have an important role in the future for the detection of prostate pathologies in neutered dogs.

**Abstract:**

Prostatic neoplasia (PN) occurs in 5–7% of dogs with prostatic disease, with castrated dogs having the same or higher prevalence when compared to intact dogs. Considering the promising results achieved by performing contrast-enhanced ultrasound (CEUS) in intact dogs to detect PN, the present study aimed to acquire data on the prostatic perfusion pattern in neutered dogs. CEUS was performed in 64 neutered dogs, using a 5–7.5 MHz linear transducer with coded harmonic capability, dedicated analytical software, and a second-generation contrast agent, SonoVue. After B-mode evaluation was performed to assess mean prostate volume, the CEUS examination was undertaken. The flow of contrast agent was visible 10 s after injection. The subcapsular vessels were highlighted and produced rapid peripheral rim enhancement. Subsequently, the contrast agent reached the prostatic urethra via the parenchymal arterioles and gradually reached the entire prostate. Perfusion peak intensity (PPI) and time to peak (TTP) values were respectively 45.3% and 34.1 s. The measured parameters were compared with those obtained in previous studies on intact dogs with normal and with pathological patterns. In this study, CEUS showed features that may be promising for its use as a diagnostic tool for early detection of PN in neutered dogs.

## 1. Introduction

Prostate neoplasia (PN) is a rare disease (prevalence of 0.43%) in both intact and neutered dogs [1], with Shetland Sheepdogs and Scottish Terriers at increased risk [2]. The mean age of diagnosis is 10 years. Adenocarcinoma, transitional cell carcinoma, and undifferentiated carcinoma are the most frequently reported PN [3,4,5,6].

A higher prevalence of PN is seen in neutered dogs; however, both neutered and intact animals develop it at the same age. This aspect suggests that neutering is not an initiator of cancer, but promotes tumor progression [2,6], or at least does not protect against the development of neoplasia.

Neutering is a routine surgical procedure in dogs and results in a decrease in testosterone and its active metabolite DHT (dihydrotestosterone) in the general circulation. The reduction of these hormones leads to prostate shrinkage and a decrease in sexual behavior, resulting in infertility [7,8]. In the last two decades, several studies have shown a statistically significant increased risk of diagnosis of various neoplastic diseases in neutered males and females compared to intact dogs, which has sparked an international debate on whether elective neutering is still a tool that should be routinely performed [9,10]. However, a definitive explanation of how the absence of gonadal hormones may influence the development of neoplasia in reproductive or nonreproductive tissues is still pending [11].

Early diagnosis of PN in dogs remains challenging, because of the lack of specific tumor markers found in tissue, blood, bone marrow, or other body fluids; therefore, the true incidence of prostate cancer may be higher than currently thought [6].

The first step in a PN diagnosis is digital rectal examination, which may provide information about prostate disorders [12,13]. When a prostate gland with PN is palpated, it may be felt as an oversized, irregular, immobile, asymmetric mass that may occasionally cause pain when touched [14]. In normal dogs, surgical castration results in a 70% reduction in the size of the gland. Although this process begins as early as 7–14 days after neutering, complete regression of the gland can be expected by 4 months [7]. Nevertheless, the assessment of prostate size may not be useful to differentiate between malignant and benign prostate conditions [15].

On B-mode ultrasound, the prostate may appear increased in dimension and asymmetrical, with irregular and poorly defined outlines. Focal or diffuse, hyperechoic, or mixed lesions may be found throughout the parenchyma. Hyperechoic foci with acoustic shadowing representing mineralization and cavitary, cyst-like lesions varying in size, shape, and number may also be present. Extension of pathologic changes to the urethra or bladder neck, enlargement of regional lymph nodes, and disruption of the capsule are threatening ultrasound signs suggestive of neoplasia [5,16,17]. Interestingly, neutered dogs with prostatic mineralization are likely to have PN, unlike intact male dogs with prostatic mineralization, which may be affected by either neoplastic or non-neoplastic prostatic disease [15].

Histological evaluation by ultrasound-guided fine needle aspiration (FNA) has been shown to be the most accurate test for detecting PN. Nevertheless, due to the low contrast and specificity existing between malignant areas and normal tissues on ultrasound images, nontargeted 12-site systematic biopsies covering the whole prostate gland are often performed to detect neoplasia, causing unnecessary pain to the patient [18,19,20]. Therefore, due to the lack of specific tumor ultrasonographic patterns, it is of great importance to develop innovative imaging techniques to increase the sensitivity and specificity of prostate cancer screening at an early stage, which may become very helpful to collect fewer and targeted samples, thereby reducing pain and stress.

The staging of canine PN should include a computed tomography scan, which may potentially be performed before cytological or histological results are available [20,21]. Magnetic resonance imaging and scintigraphy are rarely performed [4,22].

In recent years, new advanced ultrasound techniques have been undertaken for the detection of prostate cancer in dogs, such as color and pulsed Doppler sonography, contrast-enhanced ultrasound (CEUS) [23], and elastography [24].

In veterinary medicine, CEUS has been used successfully to accurately detect the perfusion patterns of the liver, spleen, and kidneys in healthy dogs [24,25] and showed some differences between malignant and benign hepatic, renal, and splenic nodules in dogs and cats as a function of perfusion patterns [26,27].

CEUS is a technique that uses a specific contrast agent injected into the vessels, to highlight the small vascular bed. The contrast agent mainly constitutes microbubbles consisting of a gas core and a stabilized biological shell [28]. Despite there being few studies about CEUS, this method may be performed to collect detailed information about lesions and to distinguish a benign lesion from a malignant one, by evaluating prostate gland vascularization [28,29]. Unlike color Doppler sonography, CEUS allows measurement of tissue perfusion independently of vessel size and vascular flow velocity. Perfusion can be assessed quantitatively, using replenishment kinetics or derivates thereof. Tumor perfusion is a surrogate parameter for their viability and, moreover, the degree of vascularity of a tumor can express its aggressiveness and help to predict its response to treatment [30].

Second-generation contrast agents, such as SonoVue^®^ (Bracco Diagnostics, Inc., Milan, Italy) and Sonazoid^®^ (Daiichi-Sankyo Co., Ltd., Tokyo, Japan), are now commercially available in North America, Europe, and Asia. SonoVue^®^, consisting of sulfur hexafluoride (SF_6_) within a phospholipid shell, allows visualization of small vessels and microvascular perfusion in capillary beds and tumors [31].

Images can be directly observed and show vascular detail or may be subjected to quantitative analysis of specific regions of interest using commercial software, allowing assessment of perfusion peak intensity, time to peak, persistence within the vascular bed, and time for complete clearance of the contrast agent [32].

In recent years, several European research groups have reported promising results of CEUS for the diagnosis of PN and for guiding biopsies both in human and veterinary medicine.

According to a human study by Halpern et al. [33] in 2012, there are significant benefits of CEUS compared to a systematic biopsy in distinguishing aggressive PN. 

Mitterberger and colleagues reported a higher detection rate of prostate cancer in humans using CEUS along with Doppler during systematic biopsy [34].

In addition, Frauscher et al. [35] reported a significant increase in the detection rate of human prostate cancer with CEUS, with a 2.6-fold higher probability than biopsy of detecting prostate cancer. 

Considering the excellent results of CEUS in human medicine, its application has also recently increased in veterinary medicine.

The work by Russo et al. [36] described the normal perfusion pattern and perfusion dynamics of the dog prostate, using CEUS. 

Interestingly, PN in intact dogs can indeed be detected with CEUS, and there are trends in perfusion parameters between tumor types. Peak intensity values of perfusion (PPI) and time to peak (TTP) values are higher in prostatic carcinoma than in leiomyosarcoma. In cases of prostatic carcinoma, there is hyper-perfusion of the tumor during the wash-in phase and hypo-echogenicity during the wash-out phase, when compared to normal dogs. Leiomyosarcoma can be characterized in all phases by a homogeneous anechoic nonperfused area with surrounding highly vascularized parenchyma [23,32].

The purpose of the present study was to describe the normal perfusion pattern and perfusion dynamics of prostate gland in castrated dogs with contrast-enhanced ultrasound and to consider whether this technique might be applicable for the detection of PN in this group.

## 2. Materials and Methods

Sixty-four, clinically normal, adult male crossbreed dogs, neutered at least 6 months prior to the present study, with an average age of approximately 2 to 14 years old and weighing between 7 and 55 kg, were used in this work.

Consecutive patients were prospectively enrolled from the “Zia Giuseppina” kennel in Caivano (Naples, Italy), assisted by two veterinary officers, in charge of the health control of the kennel. All experimental protocols in accordance with the relevant guidelines and regulations were approved by the Ethics Committee of the Department of Veterinary Medicine and Animal Production of the University of Naples, Federico II (protocol code 2016/0090751).

The dogs recruited for the present study underwent a standardized protocol that included a clinical examination, serum chemistry profile, complete blood cell count, and urinalysis (data not shown), as well as B-mode and CEUS ultrasound examination of the prostate. A 20G intravenous, three-way valved catheter was placed in the cephalic vein to rapidly infuse the bolus dose of freshly prepared contrast agent. The dogs were positioned in right lateral recumbency and linear (4–18 MHz) and microconvex transducers (6.6–8.0 MHz - Esaote Mylab 30 gold, Genova, Italy) were used to obtain scans of the prostate gland. The prostate and iliac lymph nodes were evaluated on the basis of their size, shape, margins, echogenicity, echotexture, and capsular integrity.

All patients were cooperative and, thus, no sedative or anesthesia was needed.

The transducer was placed at the level of the bladder neck and then moved, caudally, to visualize the prostate, in the longitudinal plane until a detailed image of the gland was obtained. The image was then frozen, and the maximal length (L) from the cranial to the caudal pole of the prostate and the maximal depth (DL) from the dorsal to the ventral part of the prostate were measured. Then, the transducer was rotated through 90° to obtain a transverse section.

The maximum width (W) and depth (DT) were measured, using the Atalan formulas (Atalan et al. [37]).

The prostate volume (PV) and the prostate weight (PW) were also calculated using the Atalan formulas [37].

PV = 0.487 × L × W × (DL + DT)/2 + 6.38.(1)

PW = 0.508 × L × W × (DL + DT)/2 + 3.21.(2)

Data were compared using linear regression analysis with other parameters including body weight, age, and age at castration, to establish potential relationships, as described by previous studies in intact dogs [37,38]. Color Doppler examination was performed at a frame rate of 0.7–1.4 frames per second, to study the prostate gland vascularization. The probe was then moved dorsally and cranially, to evaluate medial iliac lymph nodes by B-mode ultrasound.

For the CEUS examination, a second-generation contrast agent SonoVue^®^ (sulfur hexafluoride microbubbles; Bracco Imaging S.p.A., Milan, Italy) was used in combination with a 5–7.5 MHz linear transducer with coded harmonic capability and dedicated software for contrast-enhanced ultrasound analysis (Contrast Tuned Imaging (CnTI-TM), Contrast Tuned Imaging technology, Esaote, Genova, Italy).

To decrease the acoustic impact of the ultrasound waves on the microbubble contrast agent and to increase its persistence in the blood flow, the mechanical index was always less than 0.1 (range 0.05–0.1), corresponding to an acoustic pressure lower than 45 kPa. A single focal zone was placed in the deepest part of the prostate. The overall gain and time gain compensation were adjusted, so that no signal from the prostatic parenchyma was present and only a very low background signal from the prostatic capsule was retained, to have an anatomical reference in the image.

The contrast agent was injected into the cephalic vein at a dose of 0.03 mL/kg of prepared solution (5 mg/mL) followed by a rapid bolus of 5 mL of saline solution. The timer was activated at the moment of the start of the injection (T = 0), and the flow of the contrast agent into the prostate was observed in real time.

Care was taken to keep the probe in the same position for at least 60 s.

Difficulties occurred in the case of particularly obese dogs, with BCS (Body condition score) > 4, where the prostate was partly or entirely within the pelvic cavity, and the operator had to increase the probe pressure to clearly visualize the prostatic margins and keep the prostate still in the center of the ultrasound image, for the whole time of the examination (60 s).

The entire examination was digitally recorded, to be reviewed, so that the enhancement pattern could be analyzed.

A commercial software application, Qontrast^®^ (EC mark nr.0051, class IIA, Bracco, Milan, Italy) was used to design time–intensity curves. To obtain information, one frame was selected every 1 s for the first 60 s of the videos. In each sampled frame, the entire prostate was used as a single region of interest (ROI), manually defined by drawing a line around the shape of the prostate. A contrast-enhanced time–intensity curve was calculated for the ROI. The software calculated time–intensity curves on a pixel-by-pixel basis, fitted them to parametric curves, and calculated the following parameters, starting from T0: PPI (perfusion peak intensity) expressed as a percentage, TTP (time to peak, starting from T = 0) expressed in seconds, MTT (medium transit time) expressed in seconds, RBV (regional blood volume) expressed in cm^3^, and RBF (regional blood flow) expressed in cm^3^/s. Mean values and standard deviations of the previous parameters were then calculated.

Patients were monitored for 2 h after the contrast agent injection, to control and prevent any side-effects. The whole examination lasted at least 15 min in each dog. There were eight examinations per day, and they were performed once a week for 8 weeks, in a total of 64 patients.

Repeated-measures ANOVA was performed to understand whether there was a difference in PPI percentage values and TTP values at different time points (15 s, 30 s, and 45 s). Greenhouse–Geisser correction was used when Mauchly’s test of sphericity was violated. When significant interaction effects were observed, post hoc pairwise comparisons with Bonferroni adjustment were applied to discover which specific means differed.

All analyses were performed using SPSS software version 17.1 for Windows (SPSS Inc., Chicago, IL, USA), and a *p*-value <0.05 was considered statistically significant.

## 3. Results

No physical, laboratory, or ultrasonographic abnormalities and no side-effects or anaphylactic reactions related to the contrast-enhanced ultrasound examination were found in any of the 64 examined dogs.

**Figure 1:**
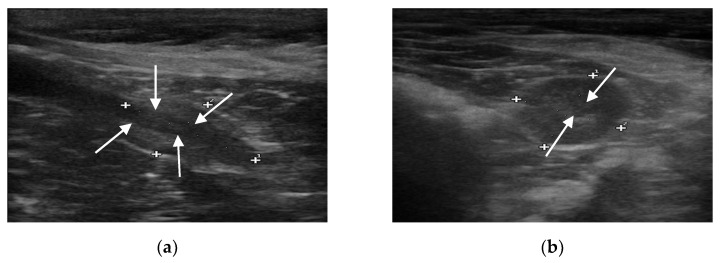
B-mode images of the prostate gland (case number 56). (**a**) Longitudinal view of the prostate gland, which appears oblong in shape, mildly echogenic, compared to the hypoechoic urethra (evidenced with arrows). (**b**) Transverse view of the same patient, in which the prostate appears ovoidal in shape and, in its center, the hypoechoic circular urethra is visible (evidenced with arrows).

B-mode ultrasound revealed that all the prostate glands had an oval shape with clear and smooth margins, as well as homogeneous and hypoechoic parenchyma. The urethra was always visible as anechoic to hypoechoic linear (in longitudinal view) and rounded (in transverse view) area, surrounded by a hyperechoic wall, which was not always visible (Figure 1).

No focal lesions were discovered in the prostatic parenchyma, and the sizes of the iliac lymph nodes were within normal limits.

**Figure 2:**
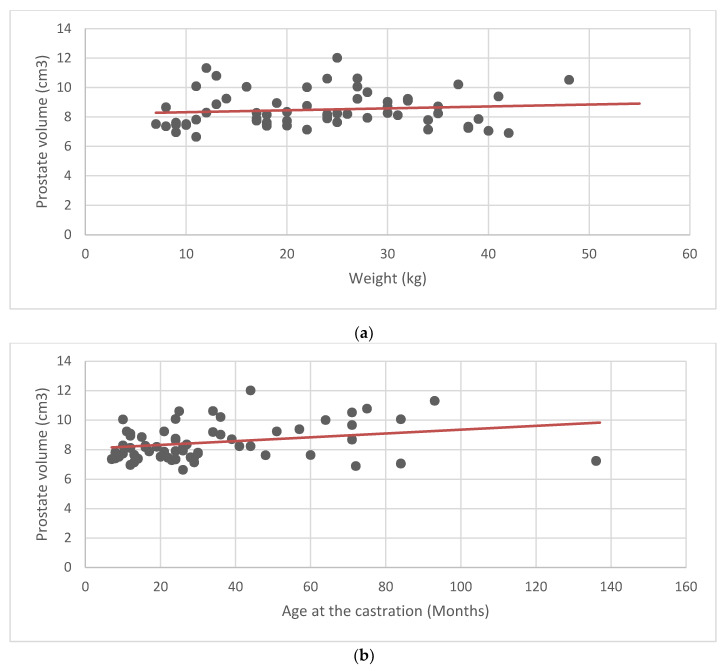

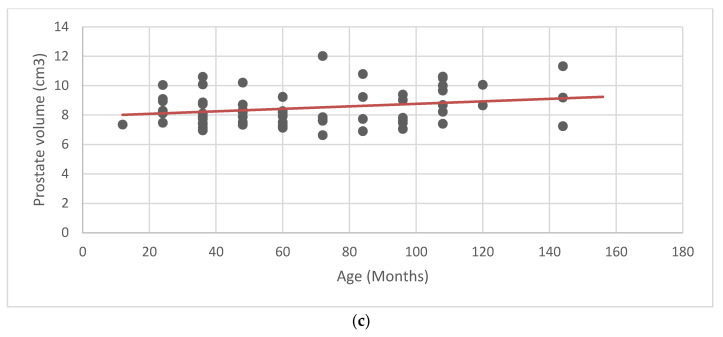
Linear regression analysis was performed to describe a hypothetical correlation between prostate volume and other parameters. None of the examined parameters seemed to influence the prostate volume in the 64 castrated dogs: (**a**) relationship between prostate volume and weight; (**b**) relationship between prostate volume and age at castration; (**c**) relationship between prostate volume and age.

According to the measured parameters, the average prostate volume was 8.48 cm^3^ ± 1.19 (±SD). Mean and standard deviation were calculated for the prostate volume, weight, age, and age at castration (Table 1). Scatter plots and linear regression analysis were performed to look for a possible correlation between the average values of the prostate volume, and the weight, age, and age at castration (Figure 2). No obvious relationship between prostate volume and the other parameters was found.

**Figure 3:**
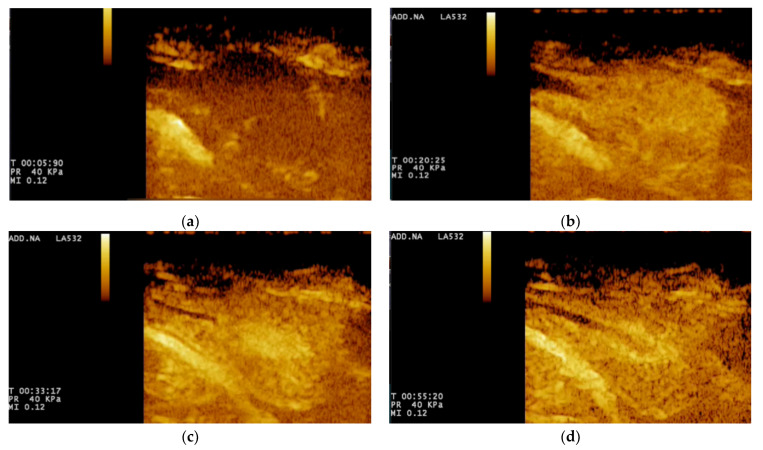
Contrast-enhanced ultrasound examination: (**a**) frame at 5 s from T0. The contrast agent has not reached the prostate yet, therefore, the gland appears to be still hypo-enhanced; (**b**) frame at 20 s from T0. The contrast agent is increasing the gland echogenicity; (**c**) frame at 33 s from T0. The wash-in phase ends up with a peak enhancement. The prostate resulted to be hyper-enhanced; (**d**) frame after 55 s from T0, which represents the wash-out phase. The prostate resulted to be hypo-enhanced, with respect to the one at 33 s.

For CEUS, the wash-in phase was visible after 15 s, when there was homogeneous enhancement of the prostatic parenchyma. During the wash-out phase, a homogeneous decrease in echogenicity was seen in all cases. The urethra was constantly seen as an echogenic linear (longitudinal view) or round (transverse view) area, more intense compared to the prostatic parenchyma (Figure 3).

The flow of the contrast agent was processed using the software Qontrast^®^ (EC mark nr.0051, class IIA, Bracco, Milan, Italy), to evaluate perfusion maps and time–intensity curves, and the results are shown in Table 2.

A repeated-measures ANOVA with Greenhouse–Geisser correction determined that mean values of PPI percentages differed statistically significantly between time points (F = 6.00, *p* < 0.01). Post hoc tests using the Bonferroni correction revealed that there were no significant differences between the PPI average values described at 15 s and those at 45 s (*p* = 0.23), nor between the average PPI values at 30 s and those at 45 s (*p* = 0.51).

**Figure 4:**
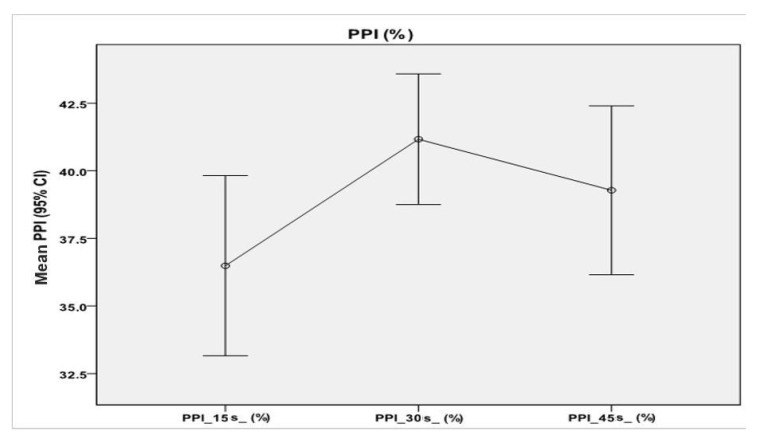
Mean and 95% confidence intervals for perfusion peak intensity (PPI) mean values, measured at 15 s (36.49% ± 13.12%), 30 s (41.17% ± 9.53%), and 45 s (39.28% ± 12.30) from the time of injection (T0). Significant differences were detectable only when looking at the PPI mean values at 15 s and 30 s.

However, at 30 s, average PPI values increased to 41.17% ± 9.53%, which was statistically significantly different to those at 15 s (*p* < 0.001). Therefore, we can conclude that a wait of 15 s from the first measurement resulted in an increase in PPI percentage (Figure 4).

**Figure 5:**
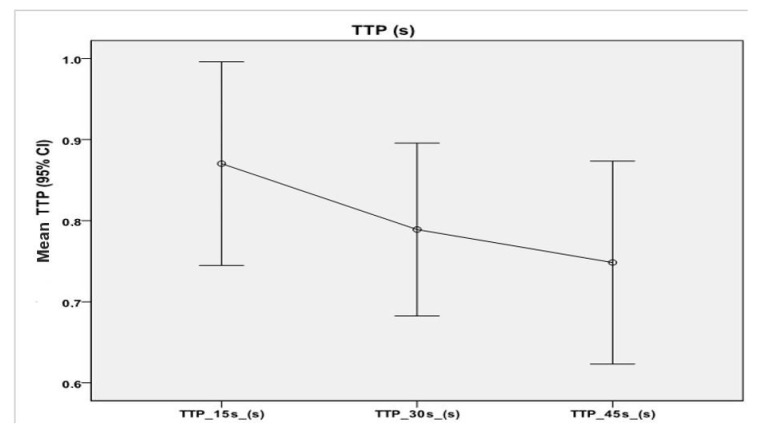
Mean and 95% confidence intervals for time to peak (TTP) mean values, measured at 15 s (0.87 ± 0.49 s), 30 s (0.79 ± 0.42 s), and 45 s (0.75 ± 0.49) from the time of injection (T0). No significant differences among all time points were detectable.

The same statistical analysis was performed in order to find significant differences at the three time points for TTP mean values (Figure 5), but the TTP analysis did not result significant (*p* = 0.299).

## 4. Discussion

Clinical assessment of prostate dysfunction in neutered dogs remains subjective and inaccurate. B-mode ultrasound results are particularly useful for assessing the size, shape, margins, echotexture, echogenicity, and position of the prostate gland, as well as for evaluating the draining reproductive lymph nodes. Assessment of prostate size is an important component in differentiating prostate disease in dogs and is usually performed by rectal palpation, abdominal radiographs, and ultrasound [39]. Although it seems to be relatively easy to determine prostate size using linear measurements of prostate height, width, or length, or to estimate prostate volume [39,40,41], there is still debate about the exact relationship between the prostate gland size and the canine body weight in intact dogs [37,38], possibly due to the potential complication of age-related changes in prostate volume [42,43]. In the work by Atalan et al. [37], it was demonstrated that prostatic weight and volume were related to body weight and age in intact dogs but not in neutered dogs. However, since prostate volume was only measured in two neutered dogs in that study, no statistical analyses were performed on prostate volume and other parameters in this group.

Orchidectomy causes a significant reduction in the prostatic dimension, beginning soon after 7–14 days, and reaching 70% of the entire volume of the gland in 6 months [7]. Nonetheless, none of the previous studies provided a reference range for prostate volume in neutered dogs. In the present study, we found that prostate size did not appear to be related to body mass in neutered dogs, and the average of the prostatic volume was 8.48 cm^3^ ± 1.19 (±SD).

To determine the normal range of prostatic volume found in healthy neutered dogs, it was important to examine a cohort of 64 adult castrated dogs (age 2 to 14 years and weight 7–55 kg) with no clinical signs of prostatic diseases. There was no apparent correlation between weight, age, and age at castration, and prostate volume, suggesting that, in neutered dogs, the prostate volume is not influenced by any of the previous parameters. Nonetheless, due to the low sample size, further studies are needed on a larger number of castrated dogs, to establish a recognized reference range.

The prostate may succumb to both focal and diffuse parenchyma changes, which may be hyperechoic, hypoechoic, or of mixed echogenicity. These changes are also nonspecific and require further investigation, which may include Doppler or contrast-enhanced ultrasound imaging [32]. Color Doppler evaluation appears to be useful for the assessment of the vascularity in intact dogs, but it is unsuccessful in neutered dogs because of the reduced prostate vessel diameter. Flow within intratumoral neovessels (10–40 μm diameter) may also be below the resolution of conventional Doppler ultrasound. This hypothesis could be supported by our study, where, despite using low PRF (pulse repetition frequency), which increases the detection of slow vascular flow, the prostatic artery and its branches could not be seen.

CEUS, however, allows the visualization of microvascularization, making it an interesting and promising diagnostic tool for the evaluation of the prostate especially within the castrated dogs, where vessel size is smaller than that in intact ones. In human medicine, CEUS is a well-established technique for the diagnosis of PN. A study by Unal et al. [44] described that CEUS detected 68–79% of all tumor foci larger than 5 mm, leading the authors to conclude that CEUS was the best single diagnostic tool for prostate carcinoma detection. Our investigation produced detailed prostatic perfusion patterns, which can serve as a normal dataset of prostatic appearance in neutered dogs and which can possibly be used as an early diagnostic method for detecting focal lesions related to PN.

Real-time contrast study allowed a better and detailed visualization of the prostatic gland vascularization than color Doppler examination. Nonetheless, since the qualitative analysis results are subjective, to perform an objective evaluation of the perfusion kinetics, collected data were standardized with a dedicated contrast-enhanced ultrasound analytical software (Q-contrast, Bracco, Italy), allowing the collection of all parameters obtained by perfusion maps and time–intensity curves. The most significant perfusion parameters were PPI (perfusion peak intensity) and TTP (time to peak). In particular, the PPI average was 45.2% (±8.2%), while TTP average was 34.1 s (±7.9 s). Intact dogs had a PPI average of 16.8% and a TTP average of 33.6 s [36]. Vignoli et al. [23] evidenced that there were no significant differences in prostatic perfusion in dogs with benign prostatic pathology compared with normal dogs, while, in dogs affected by prostatic adenocarcinoma, the PPI average was 23.7% and the TTP average was 26.9 s, suggesting that intratumoral vessels resulted in an increased, faster enhancement of the contrast agent.

PPI calculated in neutered dogs was higher than that calculated in healthy intact dogs, while TTP values were quite similar. A possible explanation for the higher PPI value could be related to the gradual parenchymal reduction, due to the previous castration, which does not change the vascularity of the prostate, thereby increasing the intensity of the ultrasound contrast agent.

To analyze the PPI and TTP variance trends in castrated dogs, means for PPI and TTP were further calculated at three different time points. Some necessary adjustments to the analysis models were included, to take into account specific features of the population. In particular, Greenhouse–Geisser correction (GGc) adjusts for the lack of sphericity in repeated-measures ANOVA. In this case, the *p*-value results were lower than 0.005. Taking multiple samples, then, the PPI in castrated dogs was coherent 99% of the time with the results found in our work, in contrast to TTP, whose *p*-value was 0.299, showing no significance of the coefficients. Further analysis was conducted to verify the existence of significant differences at the three time points. Results were significant when looking at the difference between 15 and 30 s. Interestingly, coefficients for PPI at 30 and 45 s were not statistically different. Lastly, TTP parameters showed no significant differences across all time sets. Further studies on a higher number of samples should be performed to standardize PPI and TTP parameters in the prostate of castrated dogs.

In the present study, none of the 64 examined dogs showed any focal or multifocal lesions related to PN, despite the literature reporting a higher incidence in neutered dogs. It has been reported that targeted biopsies during CEUS increase the detection rate of human prostate cancer [45]. In neutered dogs, we believe that targeted biopsies during CEUS can be used to reduce the number of core biopsies and to establish clear diagnostic samples.

## 5. Conclusions

In conclusion, CEUS seems to be a sensitive diagnostic tool compared to B-mode ultrasound in the analysis of prostatic features in castrated dogs.

The continued advances made in diagnostic imaging technology have led to substantial improvement in disease investigation, and it appears that contrast-enhanced ultrasound imaging in particular will significantly improve our ability to diagnose prostatic pathology in neutered dogs.

## Figures and Tables

**Table 1:** Mean and standard deviation of the reported parameters in the 64 dogs.

Statistical Variables	Weight (kg)	Age (Months)	Age at Castration (Months)	Prostate Volume (cm^3^)
Mean	23.72	67.88	35.02	8.48
Standard Deviation	10.69	35.60	28.55	1.19

**Table 2:** Mean and standard deviation of the CEUS parameters calculated using the Q-contrast software in the prostates of the 64 examined dogs. PPI, perfusion peak intensity; TTP, time to peak; MTT, medium transit time; RBV, regional blood volume; RBF, regional blood flow.

Statistical Variables	PPI (%)	TTP (s)	RBV (cm^3^)	RBF (cm^3^/s)	MTT (s)
Mean	45.29	34.07	3192.62	56.19	54.26
Standard Deviation	8.19	7.93	1723.20	13.78	21.35

## Data Availability

The data presented in this study are available on request from the corresponding author.

## References

[1] Axiak S.M., Bigio A. (2012). Canine prostatic carcinoma. Compend. Contin. Educ. Vet..

[2] Bryan J.N., Keeler M.R., Henry C.J., Bryan M.E., Hahn A.W., Caldwell C.W. (2007). A population study of neutering status as a risk factor for canine prostate cancer. Prostate.

[3] Cornell K.K., Bostwick D.G., Cooley D.M., Hall G., Harvey H.J., Hendrick M.J., Pauli B.U., Render J.A., Stoica G., Sweet D.C. (2000). Clinical and pathologic aspects of spontaneous canine prostate carcinoma: A retrospective analysis of 76 cases. Prostate.

[4] LeRoy B.E., Northrup N. (2009). Prostate cancer in dogs: Comparative and clinical aspects. Vet. J..

[5] Smith J. (2008). Canine prostatic disease: A review of anatomy, pathology, diagnosis, and treatment. Theriogenology.

[6] Teske E., Naan E.C., van Dijk E.M., van Garderen E., Schalken J.A. (2002). Canine prostate carcinoma: Epidemiological evidence of an increased risk in castrated dogs. Mol. Cell. Endocrinol..

[7] Johnston S.D., Kamolpatana K., Root-Kustritz M.V., Johnston G.R. (2000). Prostatic disorders in the dog. Anim. Reprod. Sci..

[8] Romagnoli S. Surgical gonadectomy in the bitch and queen: Should it be done and at what age. 2008. Proceedings of the Southern European Veterinary Conference and Congreso Nacional AVEPA.

[9] Sorenmo K.U., Goldschmidt M., Shofer F., Goldkamp C., Ferracone J. (2003). Immunohistochemical characterization of canine prostatic carcinoma and correlation with castration status and castration time. Vet. Comp. Oncol..

[10] McKenzie B. (2010). Evaluating the benefits and risks of neutering dogs and cats. Cab Rev. Perspect. Agric. Vet. Sci. Nutr. Nat. Resour..

[11] Schrank M., Romagnoli S. (2020). Prostatic neoplasia in the intact and castrated dog: How dangerous is castration?. Animals.

[12] Weaver A. (1981). Fifteen cases of prostatic carcinoma in the dog. Vet. Rec..

[13] Hargis A.M., Miller L.M. (1983). Prostatic carcinoma in dogs. Compend. Contin. Educ. Pract. Vet..

[14] White R.A.S. (2000). Prostatic surgery in the dog. Clin. Tech. Small Anim. Pract..

[15] Bradbury C.A., Westropp J.L., Pollard R.E. (2009). Relationship between prostatomegaly, prostatic mineralization, and cytologic diagnosis. Vet. Radiol. Ultrasound.

[16] Mattoon J.S., Nyland T.G., Mattoon J.S., Nyland T.G. (2015). Prostate and Testes. Small Animal Diagnostic Ultrasound.

[17] Russo M., Vignoli M., England G.C.W. (2012). B-mode and contrast-enhanced ultrasonographic findings in canine prostatic disorders. Reprod. Domest. Anim..

[18] Cunto M., Mariani E., Anicito Guido E., Ballotta G., Zambelli D. (2019). Clinical approach to prostatic diseases in the dog. Reprod. Domest. Anim..

[19] Thiemeyer H., Taher L., Schille J.T., Harder L., Hungerbuehler S.O., Mischke R., Hewicker-Trautwein M., Kiełbowicz Z., Brenig B., Schütz E. (2019). Suitability of ultrasound-guided fine-needle aspiration biopsy for transcriptome sequencing of the canine prostate. Sci. Rep..

[20] Liu C., Xing M., Cong B., Qiu C., He D., Wang C., Xiao Y., Yin T., Shao M., Qiu W. (2019). In vivo transrectal imaging of canine prostate with a sensitive and compact handheld transrectal array photoacoustic probe for early diagnosis of prostate cancer. Biomed. Opt. Express.

[21] Ravicini S., Baines S.J., Taylor A., Amores-Fuster I., Mason S.L., Treggiari E. (2018). Outcome and prognostic factors in medically treated canine prostatic carcinomas: A multi-institutional study. Vet. Comp. Oncol..

[22] Lévy X., Mimouni P., Loukeri S., Claret E. Canine prostate specific esterase as a diagnostic marker for BPH: Validation study of the in-clinic test. Proceedings of the Oral exposition in International Congress EVSSAR 2017.

[23] Vignoli M., Russo M., Catone G., Rossi F., Attanasi G., Terragni R., Saunders J.H., England G.C. (2011). Assessment of Vascular Perfusion Kinetics Using Contrast-enhanced Ultrasound for the Diagnosis of Prostatic Disease in Dogs. Reprod. Domest. Anim..

[24] Domosławska A., Zduńczyk S., Jurczak A., Janowski T. (2018). Elastography as a diagnostic tool in the prostate tumour detection in Labrador retriever. Andrologia.

[25] Ziegler L.E., O’Brien R.T., Waller K.R., Zagzebski J.A. (2003). Quantitative contrast harmonic ultrasound imaging of normal canine liver. Vet. Radiol. Ultrasound.

[26] Nakamura K., Sasaki N., Yoshikawa M., Ohta H., Hwang S.J., Mimura T., Yamasaki M., Takiguchi M. (2009). Quantitative contrast-enhanced ultrasonography of canine spleen. Vet. Radiol. Ultrasound.

[27] O’Brien R.T., Iani M., Matheson J., Delaney F., Young K. (2004). Contrast harmonic ultrasound of spontaneous liver nodules in 32 dogs. Vet. Radiol. Ultrasound.

[28] Hunt D., Romero J. (2017). Contrast-Enhanced Ultrasound. Magn. Reson. Imaging Clin. N. Am..

[29] Bennett T.C., Matz B.M., Henderson R.A., Straw R.C., Liptak J.M., Selmic L.E., Collivignarelli F., Buracco P. (2018). Total prostatectomy as a treatment for prostatic carcinoma in 25 dogs. Vet. Surg..

[30] Delorme S., Krix M. (2006). Contrast-enhanced ultrasound for examining tumor biology. Cancer Imaging.

[31] Tamura M., Nakamura K., Osuga T., Shimbo G., Sasaki N., Morishita K., Ohta H., Takiguchi M. (2019). Findings of contrast-enhanced ultrasonography with sonazoid for cholangiocellular adenoma in three dogs. J. Vet. Med. Sci..

[32] de Souza M.B., da Silva L.D.M., Moxon R., Russo M., England G.C.W. (2017). Ultrasonography of the prostate gland and testes in dogs. Practice.

[33] Halpern E.J., Gomella L.G., Forsberg F., McCue P.A., Trabulsi E.J. (2012). Contrast enhanced transrectal ultrasound for the detection of prostate cancer: A randomized, double-blind trial of dutasteride pretreatment. J. Urol..

[34] Mitterberger M., Pelzer A., Colleselli D., Bartsch G., Strasser H., Pallwein L., Aigner F., Gradl J., Frauscher F. (2007). Contrast-enhanced ultrasound for diagnosis of prostate cancer and kidney lesions. Eur. J. Radiol..

[35] Frauscher F. (2007). Contrast-enhanced Ultrasound in Prostate Cancer. Eur. Oncol. Haematol..

[36] Russo M., Vignoli M., Catone G., Rossi F., Attanasi G., England G.C.W. (2009). Prostatic perfusion in the dog using contrast-enhanced doppler ultrasound. Reprod. Domest. Anim..

[37] Atalan G., Holt P.E., Barr F.J. (1999). Ultrasonographic estimation of prostate size in normal dogs and relationship to bodyweight and age. J. Small Anim. Pract..

[38] Ruel Y., Barthez P.Y., Mailles A., Begon D. (1998). Ultrasonographic evaluation of the prostate in healthy intact dogs. Vet. Radiol. Ultrasound.

[39] Barsanti J., Finco D., Ettinger S.J., Feldman E.C. (1995). Prostatic disease. Textbook of Veterinary Internal Medicine: Diseases of the Dog and Cat.

[40] Juniewicz P.E., Ewing L.L., Dahnert W.F., Hamper U.M., Dembeck C., Sanders R.C., Coffey D.S. (1989). Determination of canine prostatic size in situ: Comparison of direct caliper measurement with radiologic and transrectal ultrasonographic measurements. Prostate.

[41] Suzuki K., Ito K., Okazaki H., Ono Y., Kurokawa K., Suzuki T., Yamanaka H. (1998). Estimation of canine prostatic volume: Nomogram based on prostatic cubic volume. Int. Urol. Nephrol..

[42] Kamolpatana K., Johnston G.R., Johnston S.D. (2000). Determination of canine prostatic volume using transabdominal ultrasonography. Vet. Radiol. Ultrasound.

[43] Berry S.J., Coffey D.S., Ewing L.L. (1986). Effects of aging on prostate growth in beagles. Am. J. Physiol. Regul. Integr. Comp. Physiol..

[44] Unal D., Sedelaar J.P., Aarnink R.G., Van Leenders G.J., Wijkstra H., Debruyne F.M., De La Rosette J.J. (2000). Three-dimensional contrast-enhanced power Doppler ultrasonography and conventional examination methods: The value of diagnostic predictors of prostate cancer. BJU Int..

[45] Vignoli M. (2010). Image-Guided Biopsy and Contrast-Enhanced Ultrasonography: Alternative Methods to Improve Imaging Diagnosis. Ph.D. Thesis.

